# Methyl-GP: accurate generic DNA methylation prediction based on a language model and representation learning

**DOI:** 10.1093/nar/gkaf223

**Published:** 2025-03-28

**Authors:** Hao Xie, Leyao Wang, Yuqing Qian, Yijie Ding, Fei Guo

**Affiliations:** School of Computer Science and Engineering, Central South University, Hunan, Changsha 410000, China; College of Intelligence and Computing, Tianjin University, Tianjin, Tianjin 300350, China; Institute of Fundamental and Frontier Sciences, University of Electronic Science and Technology of China, Sichuan, Chengdu 610054, China; Yangtze Delta Region Institute (Quzhou), University of Electronic Science and Technology of China, Zhejiang, Quzhou 324000, China; Yangtze Delta Region Institute (Quzhou), University of Electronic Science and Technology of China, Zhejiang, Quzhou 324000, China; School of Computer Science and Engineering, Central South University, Hunan, Changsha 410000, China

## Abstract

Accurate prediction of DNA methylation remains a challenge. Identifying DNA methylation is important for understanding its functions and elucidating its role in gene regulation mechanisms. In this study, we propose Methyl-GP, a general predictor that accurately predicts three types of DNA methylation from DNA sequences. We found that the conservation of sequence patterns among different species contributes to enhancing the generalizability of the model. By fine-tuning a language model on a dataset comprising multiple species with similar sequence patterns and employing a fusion module to integrate embeddings into a high-quality comprehensive representation, Methyl-GP demonstrates satisfactory predictive performance in methylation identification. Experiments on 17 benchmark datasets for three types of DNA methylation (4mC, 5hmC, and 6mA) demonstrate the superiority of Methyl-GP over existing predictors. Furthermore, by utilizing the attention mechanism, we have visualized the sequence patterns learned by the model, which may help us to gain a deeper understanding of methylation patterns across various species.

## Graphical Abstract

**Graphical Abstract:**
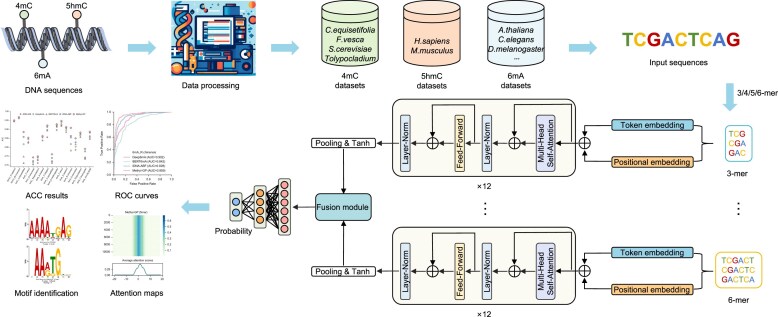

## Introduction

DNA methylation plays a crucial role in various biological processes, such as gene silencing [1], transposon silencing [2], and the regulation of chromatin organization [3]. In humans, the aberrant methylation of nucleotides typically accelerates the development of diseases, particularly cancer [4]. To better understand these mechanisms, numerous researchers have focused on the investigation of DNA methylation [5, 6]. There are three primary types of DNA methylation that have been studied: 4-methylcytosine (4mC), 5-methylcytosine (5mC), and N6-methyladenosine (6mA). Typically, 4mC and 5mC are formed by adding methyl groups to the fourth and fifth carbon atoms of cytosine (C), respectively, while 6mA involves adding a methyl group to the sixth nitrogen atom of adenosine (A). Methylation has many functions. For example, 4mC plays an important role in DNA repair and replication, protects host DNA from degradation, and regulates gene expression [7]. Generally, 5mC is regarded as an inhibitory regulator of gene expression and is related to transcriptional inhibition [8]. Moreover, 6mA plays an important role in chromosome replication, cellular defense, and cell cycle regulation [9]. Therefore, it is crucial to identify DNA methylation efficiently and accurately.

During the past several decades, biologists have extensively used next-generation sequencing (NGS), also known as high-throughput sequencing, to identify DNA methylation [10, 11]. Such as whole-genome bisulfite sequencing (WGBS) [12] and reduced-representation bisulfite sequencing (RRBS) [13]. By using NGS to analyze all enriched methylated DNA sequences, researchers can obtain high-precision information about methylation status across the entire genome, thereby facilitating a more targeted analysis of epigenetic regulation [14, 15]. However, the library construction process in NGS introduces amplification errors due to Polymerase Chain Reaction (PCR) [16], which can result in sequencing quality issues. Additionally, the read length limitation of NGS technologies (typically less than 500 base pairs) makes it difficult for them to meet the high demands of some modern biological problems, such as determining longer repetitive segments in DNA and evaluating DNA/RNA methylation modifications [17, 18]. Consequently, third-generation sequencing (TGS) technologies have been introduced, including single-molecule real-time (SMRT) technology from Pacific Biosciences [19, 20] and nanopore sequencing technology from Oxford Nanopore Technologies [21]. These technologies are capable of determining sequences with longer read lengths (up to 10 000 base pairs), eliminating the necessity to construct complex libraries, and most importantly, they do not require PCR amplification during the sequencing process.

Despite advances in NGS and TGS, these techniques are limited by specialized requirements and resources. Computational methods that leverage DNA sequence data have emerged as a complementary approach for predicting DNA methylation. These methods are primarily based on machine learning (ML) and deep learning (DL). An ML-based method utilizes feature representation algorithms to extract features from DNA sequences, and these algorithms typically rely on prior knowledge from researchers. The extract features are then fed to a classifier to make a final prediction. For example, 4mCPred [22] is a predictor designed to predict 4mC sites. 4mCPred preprocesses DNA sequences using two feature representation algorithms (position-specific trinucleotide propensity algorithm and electron-ion interaction potential algorithm), then selects the most significant subset of features to be used as input for the classifier. MM-6mAPred [23] is a method that utilizes the transition probability between adjacent nucleotides to identify 6mA sites based on a Markov model. iDNA-MS [24] is the first predictor designed to predict three different types of DNA methylation. It utilizes three different feature representation algorithms (K-tuple Nucleotide Frequency Component, Nucleotide chemical property and Nucleotide frequency, and Mono-nucleotide binary encoding) and their combinations to preprocess DNA sequences. Furthermore, iDNA-MS analyzes performance differences among various ML algorithms, including Random Forest, Naive Bayes, Bayes Net, and Decision Tree. Unlike ML-based methods that require preprocessing DNA sequences with feature representation algorithms, DL-based methods usually take DNA sequences directly as input, allowing neural networks to extract complex nonlinear relationships from sequences without human intervention. Deep6mA [25] is a robust model for predicting 6mA sites, based on convolutional neural network (CNN) and long short-term memory (LSTM) network. BERT6mA [26] uses a word2vector [27] model to encode DNA sequences into embeddings, which are then fed to a simplified BERT [28] model. iDNA-ABF [29] uses well pre-trained DNABERT models to adaptively learn features from DNA sequences and make relatively accurate predictions for various species with different methylation types.

As previously mentioned, an increasing number of researchers have proposed computational methods for predicting DNA methylation based on ML and DL. There are, however, several limitations to these methods. First, the predictive accuracy of methods for identifying methylation from sequences remains to be improved, particularly in cases with the scarcity of training samples. Second, while DL-based methods are generally more efficient than ML-based methods, some struggle to provide an intuitive representation of what the model has learned due to their limited interpretability. This limitation hinders the understanding of methylation mechanisms and patterns. Furthermore, DNA sequences themselves contain a wealth of information. However, most researchers have ignored the potential benefits of sequence patterns, often treating sequence pattern analysis and methylation identification as two separate tasks. Research has demonstrated that the same type of methylation can occur in different sequence patterns [30–33]. In plants, for example, 5mC methylation occurs in three sequence patterns: CpG, CHG, and CHH (where H = A, C, or T). In humans, the majority of 5mC occurs at CpG sites, while some are also found at sites with motifs such as CCWGG (where W = A or T). Additionally, numerous studies have noted that certain species exhibit highly similar methylation patterns [29, 34], suggesting a degree of conservation in methylation patterns among species. Naturally, two problems should be concerned: 1) Different types of methylation, and even the same type of methylation, can manifest in various sequence patterns. The ability to recognize methylation across a range of sequence patterns is crucial for the accurate prediction of different methylation patterns; 2) Different species with the same methylation type may share similar sequence patterns. It is pertinent to explore whether there is a correlation between sequence patterns and methylation prediction. Can sequence patterns be utilized to enhance model predictions?

Inspired by this, we propose Methyl-GP, a general predictor for 4mC, 5hmC, and 6mA methylation identification. Specifically, considering the diverse methylation patterns, we use four *k*-mer algorithms to tokenize DNA sequences and use a BERT-based encode module to extract features from sequences tokenized by different *k*-mer algorithms. We further fine-tuned the well pre-trained biological language models on three multi-species datasets, each corresponding to one of the three types of DNA methylation, thereby obtaining universal feature extractors for each methylation type. Next, we used these fine-tuned models to initialize the parameters of our encode module. Finally, we use a novel fusion module to integrate embeddings extracted by the encode module. Experiments on 17 benchmark datasets indicated that our model outperforms state-of-the-art methods. Moreover, ablation experiments demonstrate that fine-tuning on multi-species datasets not only reduces the requirement for large sample sizes but also enables the model to identify a broader range of sequence patterns, thereby enhancing its robustness and generalizablity. Furthermore, we employ the attention mechanism to explore local sequence patterns, uncover corresponding methylation patterns, and provide interpretable analyses of the prediction results. In summary, Methyl-GP stands out as a robust and general methylation predictor capable of accurately and adaptively identifying methylation sites.

## Materials and methods

### Benchmark datasets

**Figure 1.**
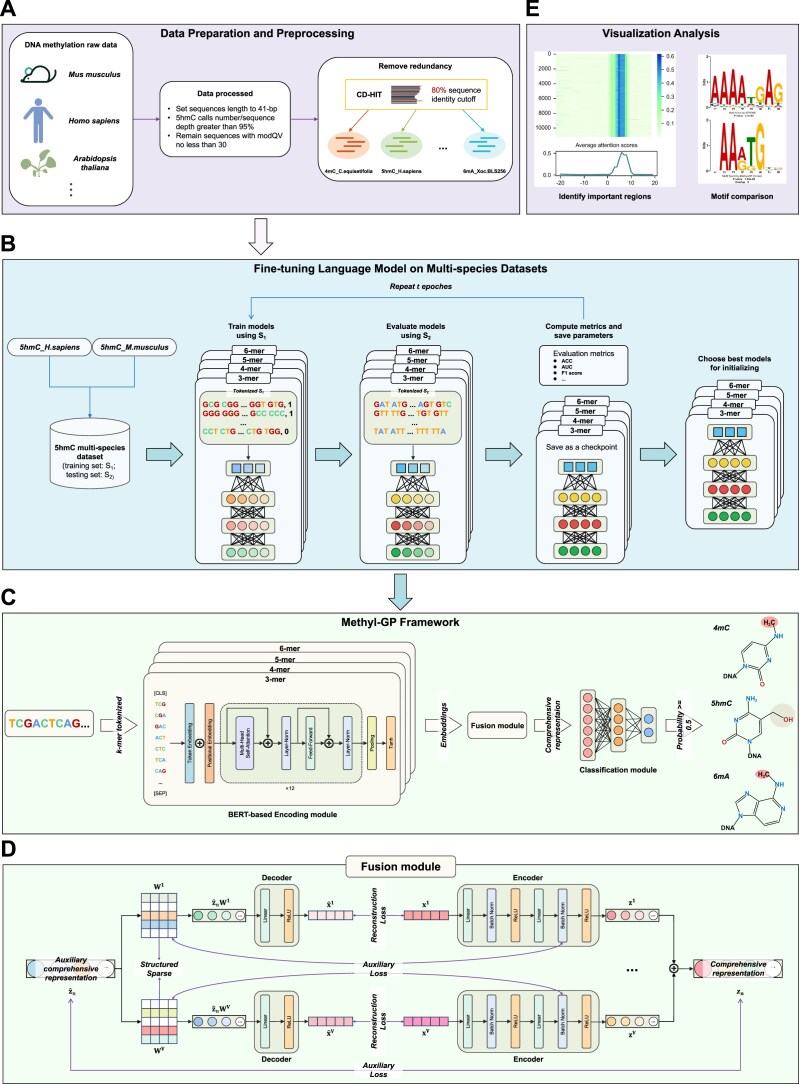
Overview of proposed Methyl-GP. (**A**) Data preparation and preprocessing involved the elimination of low-quality and redundant sequences, resulting in a total of 17 datasets including three types of DNA methylation (**B**) The process of fine-tuning language model on 5hmC multi-species dataset. (**C**) Workflow of Methyl-GP. Input DNA sequences are first tokenized using *k*-mers and then passed through the encoding module. Next, a proposed fusion module is used to integrate these obtained embeddings and outputs a comprehensive representation for classification. Finally, the classification module makes predictions based on binary classification probability values. (**D**) Architecture of the fusion module. Details are introduced in the following text. (**E**) Visualization results for interpretable analysis. Our model uses attention mechanisms to identify important regions within sequences and extract corresponding motifs.

In our study, we use the datasets originally proposed by Lv et al. [24], which contain three types of DNA methylation: 4mC, 5hmC, and 6mA. Among them, 6mA datasets comprise eleven species, including *Arabidopsis thaliana (A.thaliana)*, *Caenorhabditis elegans (C.elegans)*, *Casuarina equisetifolia (C.equisetifolia)*, *Drosophila melanogaster (D.melanogaster)*, *Fragaria vesca (F.vesca)*, *Homo sapiens (H.sapiens)*, *Rosa chinensis (R.chinensis)*, *Saccharomyces cerevisiae (S.cerevisiae)*, *Tolypocladium sp SUP5 PDA-1 (Tolypocladium)*, *Tetrahymena thermophile (T.thermophile)*, and *Xanthomonas oryzae pv. Oryzicola BLS256 (Xoc.BLS256)*. 4mC datasets consist of four species: *C.equisetifolia*, *F.vesca*, *S.cerevisiae*, *Tolypolcadium*. The 5hmC datasets include two species, *H.sapiens* and *M.musculus*. To construct these high-quality benchmark datasets, Lv et al. set all sequences with a length of 41 base pairs (bp), eliminated low-quality sequences, and removed sequences with more than 80% sequence similarity detected by the CD-HIT program (Fig. 1A) [35]. For more detailed information about the division of training sets and testing sets, as well as the counts of positive and negative samples, please refer to (Supplementary Table S1).

### Description of Methyl-GP

Methyl-GP mainly consists of three modules: a BERT-based encoding module, a fusion module, and a classification module (Fig.1C). As mentioned previously, there are many sequence patterns in different methylation types. In order to reduce information loss, Methyl-GP utilizes four BERT [28] encoders to extract embeddings from different tokenized sequences processed by *k*-mers (Fig. 1C, encode module). Then, the obtained embeddings are input into a fusion module to generate a high-quality comprehensive representation (Fig. 1D). Finally, the comprehensive representation is fed to the classification module with two fully connected layers to predict whether the input sequence is methylated or not. Additionally, we calculated the average scores of all attention heads in the last Transformer block corresponding to the input tokens and extracted the motifs based on these average attention scores. The higher the attention scores of a token, the stronger recognition ability of the model for the corresponding motif, and the more likely this motif is associated with the methylation pattern (Fig. 1E).

### BERT-based encode module

#### Encoding process of the BERT

BERT is a transformer-based [36] contextualized language representation model, and is widely used in natural language processing tasks. It introduces a paradigm of pre-training and fine-tuning, which means the model is first trained on massive amounts of unlabeled data to gain general-purpose understanding, and then fine-tuned on specific downstream tasks to better address these tasks. BERT is composed of an embedding layer and 12 Transformer blocks (Fig.1C, BERT-based Encoding module). Each block consists of a multi-head attention layer and a fully connected feed-forward layer. Additionally, residual connections and layer normalization are used around each of the two sub-layers. For a matrix *X* generated by the embedding layer, BERT performs the multi-head attention to concatenate the attention scores of multiple heads, which can be expressed as follows:


(1) \begin{eqnarray*}
MultiHead(X) = Concat(head_1, ..., head_h)W^O,
\end{eqnarray*}

where


(2) \begin{eqnarray*}
head_i = Softmax(\frac{XW_i^QXW_i^KT}{\sqrt{d_k}})\cdot XW_i^V.
\end{eqnarray*}

$X \in R^{L \times d_m}$ is the output of the embedding layer. $W^O \in R^{d_m \times d_k}$ and $\lbrace W_i^Q, W_i^K, W_i^V\rbrace _{i=0}^h \in R^{d_m \times d_k}$ are learnable parameters. *L* represents the length of the input tokenized sequences. $d_m$ is the dimension of embeddings, while $d_k$ is the dimension of *W*^*Q*^, *W*^*K*^, *W*^*V*^, and *W*^*O*^. The attention scores between tokens are computed and used as weights to sum over the rows in $XW_i^V$. This process allows each attention head $\lbrace head_i\rbrace _{i=1}^h$ to calculate the hidden states of *X*. The *MultiHead*( · ) function initially concatenates the outputs from different heads, then uses a linear layer *W*^*O*^ to ensure that the output dimension of the multi-head attention layer is equivalent to that of the embedding layer. The output of last Transformer block is then fed to a pooling layer followed by a Tanh activation function, and yields final embeddings of the tokenized sequences.

#### Pre-trained biological language model

Pre-training is crucial for enhancing the generalizability of language models; a well-executed pre-training process can largely improve the generalizability of the model. Therefore, here we use pre-trained biological language models, DNABERT [37], and fine-tune them on the multi-species datasets. The parameters of the fine-tuned model are then used to initialize the parameters of the encoding module of our Methyl-GP. DNABERT uses a human genome dataset that comprising 2.75 billion nucleotides for pre-training, thus acquiring a global and transferrable understanding of genomic DNA sequences based on up and downstream nucleotide contexts. Moreover, since the semantic information contained in DNA sequences is quite different from that of human natural language, the tokenization process of DNABERT differs significantly from that of BERT.

In BERT, a token may consist of one or several words, whereas DNABERT tokenizes a DNA sequence using *k*-mer (*k* = 3, 4, 5, 6) algorithms, which means each token consists of k bases. For example, given a DNA sequence ‘ATTAA’, it can be tokenized into a sequence of three 3-mers: ATT, TTA, TAA or into a sequence of two 4-mers: ATTA, TTAA. Therefore, for each k values, there are a total of 4^*k*^ + 5 tokens in the vocabulary, which consists of all the permutations of the *k*-mer as well as five special tokens: classification token ([CLS]), padding token ([PAD]), unknown token ([UNK]), separation token ([SEP]), and masked token ([MASK]). Methyl-GP also tokenizes DNA sequences using k-mer algorithms. As mentioned previously, we select k from 3 to 6, thereby generating four distinct embeddings corresponding to different values of k.

#### Fine-tuning language model on multi-species datasets

**Figure 2.**
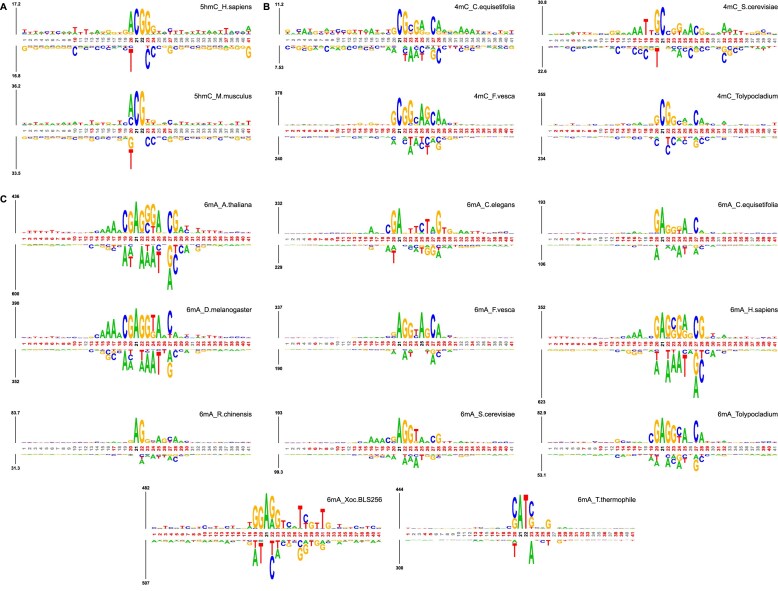
The probability-based motif logo visualized by KpLogo. (**A**)–(**C**) Motif logos of 5hmC, 4mC, and 6mA datasets, respectively. The methylated sequences in the testing set of species are used for visualizing. We set *k* = 1 to show the residue distribution at every position of different species. In general, the higher the frequency of a residue occurring at a particular position, the larger the corresponding logo.

In addition to pre-training, fine-tuning is also important for enhancing the performance of language models in downstream tasks. Finding the underlying sequence patterns surrounding the modification sites is an effective step in understanding the reasons for their modification and in revealing the biological functions of modifications [38]. Therefore, to maximize the benefits of fine-tuning and enhance the ability of the encoding module to effectively extract latent information from sequences, we first generated probability-based motif logos for all species using KpLogo (Fig. 2) [39].

For motif logos of 5hmC species, the sequence patterns are extremely similar (Fig. 2A). A highly conserved CpG (G represents guanine) motifs appear at the central region (positions 21 and 22) of the sequences, while an A frequently occurs at position 20. Moreover, we also observed an enrichment of T (thymine) in up and down-stream of 5hmC sites. As shown in (Fig. 2B), a consensus motif GCG[G/C] can be observed from position 20–23 in *4mC_C. equisetifolia*, *4mC_F.vesca*, and *4mC_Tolypocladium*. While for *4mC_S.cerevisiae*, we found a motif of AAATT in the up-stream region (position 15–19). These motifs indicated that sequence patterns are species specific; however, within *C. equisetifolia* and *F. vesca*, some conserved patterns can still be observed. As for 6mA species, it can be observed from the figures that there is a distinct motif of [G/C]AGG[C/T] [40] at positions 20–24 in most species except *T.thermophile* (Fig. 2C). For *A. thaliana*, *C. equisetifolia*, *D. melanogaster*, *F. vesca*, *H. sapiens*, *R. chinensis*, *S. cerevisiae*, and *Tolypocladium*, there is an enrichment of A and C at positions 25 and 27, respectively. For *A.thaliana*, *C.elegans*, *D.melanogaster*, *H.sapiens*, and *S.cerevisiae*, we observe a continuous AAA motif at positions 15–17. Furthermore, *A. thaliana*, *D. menalogaster*, *H. sapiens*, *S. cerevisiae*, *Tolypocladium*, and *Xoc. BLS256* all show an enrichment of T in up and downstream region of sequences. This suggests that methylation patterns among some species may be highly conserved. More importantly, for species with an insufficient samples that result in the absence of discernible sequence patterns, such as *F. vesca* and *R. chinensis*, we may leverage knowledge acquired from other species to enhance generalizability of the model.

Based on this, we first constructed three multi-species datasets based on three methylation types for language model fine-tuning: 4mC multi-species dataset, 5hmC multi-species dataset, and 6mA multi-species dataset. For example, we use *5hmC_H.sapiens* and *5hmC_M.musculus* datasets to construct 5hmC multi-species dataset (Fig. 1B). Specifically, the training (testing) set of 5hmC multi-species dataset consists of training (testing) sets from both *5hmC_H.sapiens* and *5hmC_M.musculus*. We do the same thing to construct 4mC multi-species dataset. However, due to the excessive number of samples in *6mA_T.thermophile* dataset, we excluded it when constructing 6mA multi-species dataset and fine-tuned language models solely on *6mA_T.thermophile* dataset. Since DNABERT includes a fully connected layer for classification, it can be directly applied to classification tasks. Therefore, we used common classification assessment metrics such as accuracy (ACC), area under the receiver operating characteristic curve (AUC), F1-scores to evaluate the efficacy of the fine-tuned models. We fine-tuned DNABERT models on the training set of the multi-species dataset and then evalueated the models on their testing set for 20 epochs. After each epoch, model outputs the predictive results and we save the current model parameters as a checkpoint. Given that the DNABERT models had been well pre-trained, they were able to converge rapidly during fine-tuning. Once all epochs are completed, we selected checkpoint with the highest predictive ACC as the final fine-tuned model. After fine-tuning on three multi-species datasets, we obtained DNABERT models specialized for three types of methylation, which are then used to initialize the parameters of our encoding module.

### Feature fusion module

In order to obtain the final representation of four embeddings, we propose a novel fusion module for integration. The fusion module is mainly consisted of two parts: the decoding part and the encoding part (Fig. 1D). We construct the decoding part with the aim of solving a simple task, i.e. decoding the auxiliary comprehensive representation $\widehat{z}_n$ (which should contain complete information) to the specific embedding $x_n^v$ (containing partial information). This relationship is explicitly learned through the relation matrices $\lbrace W^i\rbrace _{i=1}^V$, which means the reconstructed embedding $\widehat{x}_n^v=g^v(\widehat{z}_nW^v)$. Meanwhile, the encoding part is designed to further encode the embeddings into compact and aligned representations. However, we will encounter the out-of-sample problem when evaluating Methyl-GP on the testing set. Therefore, we use the $W^v$ and $\widehat{z}_n$ to guide the encoding process. Finally, since the representation $\lbrace z^i\rbrace _{i=1}^V$ are well aligned, we directly average them to obtain the final comprehensive representation $z_n$, and feed $z_n$ to the classification module for prediction. It is worth noting that the training process is conducted in two steps. In the first step, we train the decoding part on the training set and only update parameters of the fusion module using the SGD [41] optimizer with a learning rate of 5 × 10^−5^ and a weight decay of 3 × 10^−3^. In the second step, we train the entire model on the training set using the AdamW [42] optimizer with learning rates of 3 × 10^−5^ and 5 × 10^−5^, and weight decays of 1 × 10^−3^ and 3 × 10^−3^.

#### Decoding part

The decoding part is introduced to address a straightforward task: converting the auxiliary comprehensive representation $\widehat{z}_n$ into specific embeddings $\lbrace x_n^v\rbrace _{v=1}^V$. The decoding part consists of two components: (i) the relation matrices $\lbrace W^v\rbrace _{v=1}^V$, which quantify the relationships between $\widehat{z}_n$ and $x_n^v$; (ii) the decoder neural networks $\lbrace g^v(\cdot )\rbrace _{v=1}^V$, which learn the complex nonlinear mappings from $\widehat{z}_nW^v$ to $\widehat{x}_n^v$. The optimization target of the decoding process is:


(3) \begin{eqnarray*}
L_r = \frac{1}{N}\sum _{v=1}^V\sum _{n=1}^N\Vert x_n^v-\widehat{x}_n^v\Vert _2^2,
\end{eqnarray*}

where


(4) \begin{eqnarray*}
\widehat{x}_n^v=g^v(\widehat{z}_nW^v).
\end{eqnarray*}

Here, $\widehat{z}_n \in R^d$, $W^v \in R^{d \times h}$, *d* denotes the dimension of embeddings, and *h* represents the dimension of the hidden layer. Additionally, different embeddings contain both consistent information (shared by all embeddings) and complementary information (unique to different embeddings). For example, if the first row of $W^1$ is all zeros, this implies that the first dimension of $\hat{z}_n$ is irrelevant to the first embedding. This dimension can represent the complementary information of multiple embeddings. Conversely, if the corresponding vectors of a certain dimension are all non-zero, then this dimension is related to all embeddings, representing the consistent information of the embeddings. All the weight matrices $W^v$ are initialized randomly. To ensure that the comprehensive representation encompasses both complementary and consistent information for each embedding, we introduce structured sparse regularization for each $W^v$. This encourages certain rows of $W^v$*W*^$v$^ to become 0, indicating that the corresponding dimensions of $\widehat{z}_n$ are not associated with the $v$-th embedding:


(5) \begin{eqnarray*}
\Vert W^v\Vert _{2, 1}=\sum _{i=1}^d\Vert W_i^v\Vert _2=\sum _{i=1}^d\sqrt{\sum _{j=1}^h(w_{ij}^v)^2}.
\end{eqnarray*}

The overall loss function of the decoding part can be formulated as:


(6) \begin{eqnarray*}
\min \limits _{\lbrace W^v, g^v\rbrace _{v=1}^V, \lbrace \widehat{z}_n\rbrace _{n=1}^N} L_r + \beta \sum _{v=1}^V\Vert W^v\Vert _{2, 1},
\end{eqnarray*}

where β > 0 is the hyperparameter.

We train this part to learn an auxiliary comprehensive representation of the training data. However, when evaluating the model on new data from the testing set, we face the out-of-the-sample problem. Consequently, we introduce a new optimization target for the encoding part.

#### Encoding part

In the encoding part, we attempt to map the embeddings $\lbrace x_n^v\rbrace _{v=1}^V$ to the comprehensive representation $z_n$. The process is described as follows:


(7) \begin{eqnarray*}
z_n=\frac{1}{V}\sum _{v=1}^Vz_n^v=\frac{1}{V}\sum _{v=1}^Vf^v(x_n^v),
\end{eqnarray*}

where $f^v(\cdot)$ is the encoder neural network corresponding to the $v$-th embedding. To obtain a compact and aligned $z_n^v$, we introduce $\widehat{z}_n$ and the set of matrices $\lbrace W^v\rbrace _{v=1}^V$ to guide the learning process of encoders. Specifically, we incorporate Batch Normalization (BN) into each layer of the encoders, which helps to mitigate the covariate shift across different embeddings. Furthermore, for the final BN layer in each encoder, we regularize its weights as follows:


(8) \begin{eqnarray*}
\Vert w^v-\sigma (a\overline{w}^v+b)\Vert _2^2,
\end{eqnarray*}

where


(9) \begin{eqnarray*}
\overline{w}^v=\frac{1}{h}\sum _{i=1}^h|W_{\cdot ,i}^v|.
\end{eqnarray*}

$W^v \in R^h$ is the weight of the last BN layer of the $v$-th encoder. *a* and *b* are trainable parameters. The sigmoid function $\sigma (\cdot)$ is applied to ensure that $W^v$ lies within the interval (0,1), which promotes sparser values. In addition to $\lbrace W^v\rbrace _{v=1}^V$, we also utilize $\widehat{z}_n$ to guide the learning of $z_n$, and the auxiliary loss is defined as follows:


(10) \begin{eqnarray*}
L_a=\frac{1}{N} \sum _{n=1}^N \Vert z_n-\widehat{z}_n\Vert _2^2+\gamma \Vert w^v-\sigma (a\overline{w}^v+b)\Vert _2^2,
\end{eqnarray*}

where γ > 0 is the hyperparameter. The new optimization objective is given by the following equation:


(11) \begin{eqnarray*}
\min \limits _{\lbrace f^v\rbrace _{v=1}^V} \frac{1}{N} \sum _{i=1}^N \lambda _1 L_{ce}+\delta L_a+\lambda _2 \sum _{v=1}^V\sum _{i=1}^h |w_i^v|.
\end{eqnarray*}

Here, $L_{ce}$ represents the cross-entropy loss of the final output, while $\lambda_1$ and $\lambda_2$ denote the hyperparameters. The annealing coefficient δ is defined as $\delta=max(0,1-t/10)$, where *t* is the current training epoch. All hyperparameters of Methyl-GP when trained on the benchmark datasets are presented in (Supplemental Table S2).

### Performance assessment

We use four metrics commonly used in classification tasks to evaluate the performance of our model and other existing methods: ACC, Sensitivity (SN), Specificity (SP), and Matthews’ correlation coefficient (MCC), which can be calculated as follows:


(12) \begin{eqnarray*}
SN &=& \frac{TP}{TP+FN} \nonumber \\ SP &=& \frac{TN}{TN+FP} \nonumber \\ MCC &=& \frac{(TN\times TP)-(FN\times FP)}{\sqrt{(TN+FP)(FP+TP)(TP+FN)(TN+FN)}} \nonumber \\ ACC &=& \frac{TP+TN}{TP+FN+TN+FP}.
\end{eqnarray*}

TP, TN, FP, and FN represent the number of true positive, true negative, false positive, and false negative samples, respectively. Both ACC and MCC are used to evaluate the overall performance of the model. SN and SP represent the proportion of methylated and non-methylated samples correctly predicted by the model, respectively. In addition, we utilize the receiver operating characteristic (ROC) curve and the PR (precision-recall) curve to further compare the performance of the model. AUC and AP represent the area under the ROC curve and the PR curve, respectively [43]. Overall, the higher the values of these metrics, the better the model.

## Results

### Performance evaluation of Methyl-GP and other state-of-the-art predictors

We compared Methyl-GP with other four predictors, including iDNA-MS, Deep6mA, BERT-6mA, and iDNA-ABF. Although Deep6mA and BERT6mA are originally designed for 6mA methylation prediction only, they are the state-of-the-art DL-based methods and can easily able to predict other types of DNA methylation. On the other hand, iDNA-MS and iDNA-ABF are predictors that can be used to predict three types of methylation. Notably, since the performance of iDNA-MS and iDNA-ABF on the benchmark datasets has been reported in their original works, we use their results directly for comparison. However, complete experimental results are not available for Deep6mA and BERT6mA. Therefore, we replicated the models based on the code released by the authors and conducted experiments independently. These two models were trained on the training sets, and evaluated on the corresponding testing sets. The hyperparameters for both Deep6mA and BERT6mA are set according to the optimal configurations reported in their respective paper.

**Figure 3.**
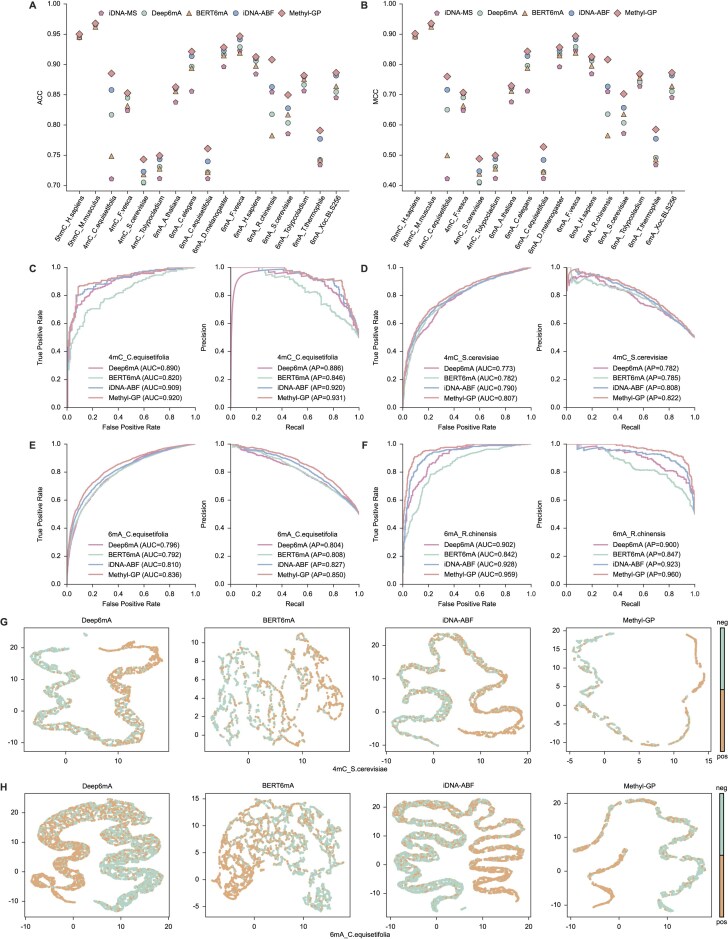
Performance comparison between Methyl-GP and other state-of-the-art predictors. (**A** and **B**) The ACC (left) and MCC (right) values of Methyl-GP and four state-of-the-art predictors on benchmark datasets. (**C**)–(**F**) Receiver operating characteristic (ROC) and precision recall (PR) curves of Methyl-GP and other three predictors in *4mC_C.equisetifolia*, *4mC_S.cerevisiae*, *6mA_C.equisetifolia*, and *6mA_R.chinensis*, respectively. (**G** and **H**) The feature space distribution visualized by UMAP for different predictors on *4mC_S.cerevisiae* and *6mA_C.equisetifolia*.

We first presented the predictive ACC (Fig. 3A) and MCC curves (Fig. 3B) of the comparison models. The detailed results of other metrics are detailed in (Supplementary Table S3). It can be observed that Methyl-GP outperforms other models on 15 out of 17 datasets. Even on *5hmC_H.sapiens* and *5hmC_M.musculus*, Methyl-GP has the same predictive ACC as the best predictors. Particularly, Methyl-GP performed better than other four predictors with a relatively large margin on five datasets: *6mA_R.chinensis*, *4mC_C.equisetifolia*, *6mA_S.cerevisiae*, *6mA_C.equisetifolia*, and *4mC_S.cerevisiae*, leading by 4.51–12.54%, 2.73–17.43%, 2.19–6.42%, 2.11–4.97%, and 2.02–3.9% in ACC, respectively. The results demonstrate that Methyl-GP is superior to other state-of-the-art predictors, particularly under conditions of data scarcity, Methyl-GP exhibits better generalizability.

Next, we plotted the ROC and PR curves for predictors on four datasets: *4mC_C.equisetifolia*, *4mC_S.cerevisiae*, *6mA_C.equisetifolia*, and *6mA_R.chinensis*, respectively (Fig. 3C–F). We can see that Methyl-GP achieves the highest AUC and AP values on four datasets. Similar results can be seen on other datasets (Supplementary Table S3), which indicate the robust performance of Methyl-GP for the generic prediction of DNA methylations. To present a more intuitive comparison of how different predictors make decisions, we use Uniform Manifold Approximation and Projection (UMAP, a widely used visualization tool) [44] to visualize the distribution of the feature representations learned by different predictors in the feature space. Two datasets are used for comparison: *4mC_S.cerevisiae* (Fig. 3G) and *6mA_C.equisetifolia* (Fig. 3H). It can be seen that the feature representations learned by BERT6mA are the most poorly distributed. Although Deep6mA and iDNA-ABF have learned distributions with clear boundaries, the distribution of samples from the same class is relatively dispersed. In contrast, Methyl-GP not only learned distributions with clear boundaries, but also clustered samples from the same class more tightly. Therefore, Methyl-GP is capable of making more accurate predictions. This demonstrates that Methyl-GP has learned a better discrimination strategy for different classes of samples, possibly due to the fine-tuning and the fusion module, which helps us discover important semantic information embedded within the sequences.

### Cross-species validation among different species

**Figure 4.**
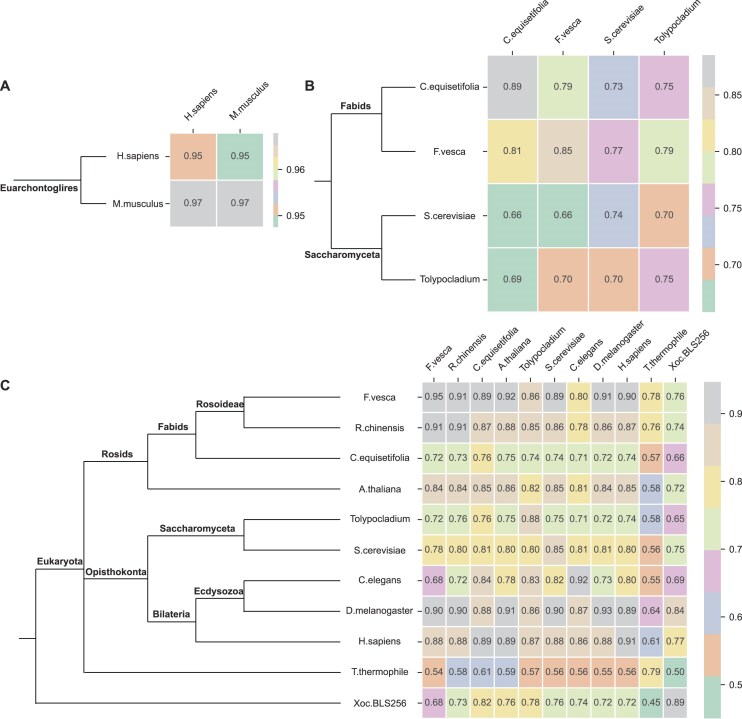
Heatmaps of cross-species validation and species phylogenetic trees. (**A**–**C**) The species phylogenetic trees (left) and predictive ACCs of cross-validation (right) of 5hmC, 4mC, and 6mA species, respectively.

To verify that sequence patterns can play a role in methylation prediction, we conducted cross-species validation to explore whether a model trained on the training set of one species can achieve satisfactory results on the testing sets of other species. To this end, we performed cross-species validation separately on 4mC, 5hmC, and 6mA datasets, and presented all predictive ACC values (Fig. 4 and Supplementary Table S4). It can be observed that the cross-species validation results of two 5hmC datasets are very close (Fig. 4A). From the perspective of sequence patterns, it can be observed that the sequence patterns of the two species are very similar (Fig. 2A). The knowledge learned by the model from the training set of one species can be effectively transferred to the testing sets of other species with similar sequence patterns. Additionally, the cross-species validation results from the 4mC datasets reveal that the transfer performance between *4mC_C.equisetifolia* and *4mC_F.vesca* is better, while the transfer performance between *4mC_S.cerevisiae* and *4mC_Tolypocladium* is superior. In contrast, the transfer performance between *4mC_C.equisetifolia* and *4mC_S.cerevisiae*, as well as between *4mC_F.vesca* and *4mC_S.cerevisiae*, is the poorest. By combining the analysis of sequence patterns, we find that the knowledge learned by the model from species with significantly different sequence patterns does not transfer well (Fig. 2B). Similar conclusions can be drawn from the results of cross-species validation on 6mA datasets. In particular, we observed that whether training the model on the training set of *T.thermophile* and then testing it on the testing sets of other 6mA species, or training the model on the training sets of other 6mA species and then testing it on the testing set of *T.thermophile*, the predictive ACC is very poor in both cases. This is more likely due to the significant difference in sequence patterns between *T.thermophile* and other 6mA species rather than the large number of samples in *T.thermophile*. In summary, cross-species validation among different species further demonstrates that sequence patterns indeed play a significant role in the prediction of methylation.

We further constructed species phylogenetic trees using Lifemap [45] to explore the relationship between methylation pattern differences and the evolutionary levels among different species (Fig. 4A–C, left). As we can see, for 5hmC species *H.sapiens* and *M.musculus*, both belong to Euarchontoglires and have a relatively close evolutionary relationship. For 4mC species, *C.equisetifolia* and *F.vesca* belong to Fabids, while *S.cerevisiae* and *Tolypocladium* belong to Saccharomyceta. This demonstrates that the evolutionary relationship between *C.equisetifolia* and *F.vesca* is closer than that between *C.equisetifolia* and *S.cerevisiae* or *Tolypocladium*. It should be noted that the sequence patterns of *C.equisetifolia* and *F.vesca* are more similar, while those of *S.cerevisiae* are more similar to *Tolypocladium*. Moreover, the model performs better in transfer training between *C.equisetifolia* and *F.vesca*, as well as between *S.cerevisiae* and *Tolypocladium*. Similarly, for 6mA species, the model performs well in transfer learning with species that have a relatively close evolutionary relationship. In contrast, the model performs poorly in transfer learning when trained or tested on *T.thermophile* and *Xoc.BLS256*, as their evolutionary relationships are very distant from those of other species. Therefore, we posit that evolutionary relationships are a significant factor influencing the sequence patterns among species.

### Fine-tuning on multi-species datasets facilitates Methyl-GP to learn more methylation patterns

**Figure 5.**
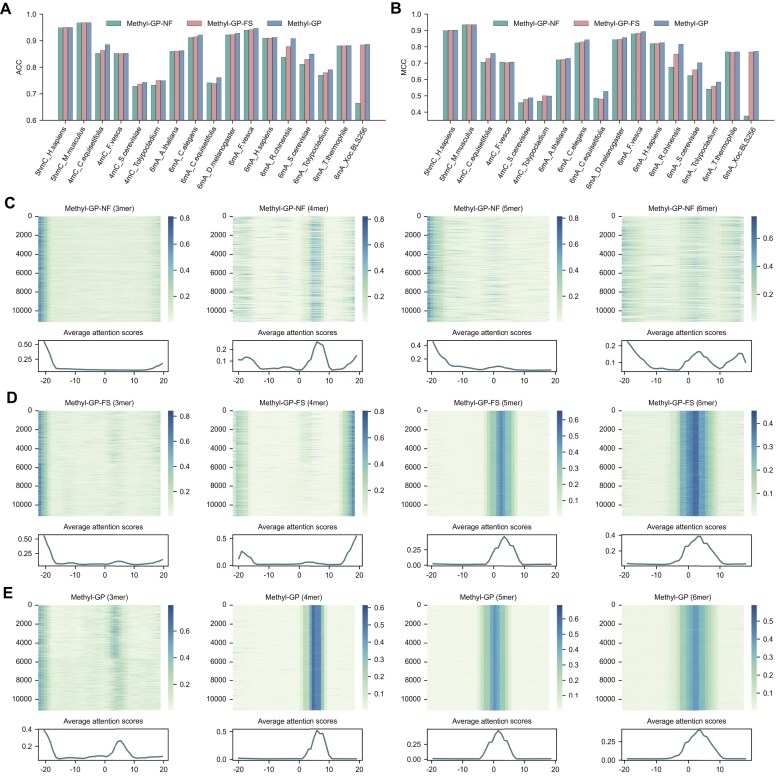
Analysis of different fine-tuning strategies. (**A**) and (**B**) The ACC and MCC values of Methyl-GP-NF (no fine-tuning), Methyl-GP-FS (fine-tuning on species specific dataset), and Methyl-GP (fine-tuning on multi-species dataset) on benchmark datasets. (**C**–**E**) The attention heatmaps for sequences in the testing set of *6mA_D.melanogaster*, obtained by the encoding module of the model corresponding to different *k*-mers.

To explore the impact of fine-tuning language model on multi-species datasets for methylation identification, we performed two ablation experiments for comparison. We used language models without fine-tuning and those fine-tuned on specific datasets to initialize the parameters of our BERT-based encoding module, respectively. For simplicity, we refer to them as Methyl-GP-NF (without fine-tuning) and Methyl-GP-FS (fine-tuning on specific datasets). We presented the ACC and MCC values of the models with three different fine-tuning strategies (Fig. 5A and B, and Supplementary Table S5). Compared to Methyl-GP-NF and Methyl-GP-FS, Methyl-GP achieved significantly improvement on *4mC_C.equisetifolia*, *6mA_C.equisetifolia*, *6mA_R.chinensis*, *6mA_S.cerevisiae*, and *6mA_Tolypocladium*. Specifically, the average ACC of Methyl-GP is 2.74% higher than that of Methyl-GP-NF and 0.80% higher than that of Methyl-GP-FS. This suggests that fine-tuning on multi-species datasets has greatly improved the predictive performance of Methyl-GP.

By calculating the attention scores based on Equation (1), we obtained a matrix representing the attention scores between all tokens. Then, we used the average attention scores from the 12 heads of the last transformer block to represent the final attention scores of all tokens. In our study, we used the model to identify whether a sequence is methylated. Specifically, the judgment is based on whether the central nucleotide (A or C) of the sequence has undergone methylation. Consequently, tokens containing the central nucleotide generally exhibit higher attention scores. Taking sequences from the testing set of *6mA_D.melanogaster* as an example, we presented the attention heatmaps of tokenized sequences processed by different *k*-mer algorithms. From the figures, we can see that Methyl-GP-NF (3-mer) does not identify any meaningful regions. Meanwhile, although Methyl-GP-NF (4-mer), Methyl-GP-NF (5-mer), and Methyl-GP-NF (6-mer) put attention upon some regions, they do not show a pronounced tendency (Fig. 5C). After fine-tuning on species-specific datasets, neither Methyl-GP-FS (3-mer) nor Methyl-GP-FS (4-mer) identifies significant regions. In contrast, Methyl-GP-FS (5-mer) and Methyl-GP-FS (6-mer) consistently put high attention upon the central regions of the tokenized sequences (Fig. 5D), implying that the model has successfully gained the ability to identify relevant methylation patterns. However, after fine-tuning on multi-species datasets, Methyl-GP (3-mer), Methyl-GP (4-mer), Methyl-GP (5-mer), and Methyl-GP (6-mer) all consistently put high attention upon the central regions of sequences (Fig. 5E), although Methyl-GP (3-mer) does not display a strong tendency. This suggests that fine-tuning on multi-species dataset indeed enhance the ability of the language models to recognize important methylation regions. Additionally, we observed that tokens in the central and adjacent downstream regions generally have higher attention scores. This indicates that the downstream regions of the methylation sites may harbor more latent information, which can help the model to make predictions.

### Combinations of *k*-mers enables Methyl-GP to identify methylation patterns adaptively

**Figure 6.**
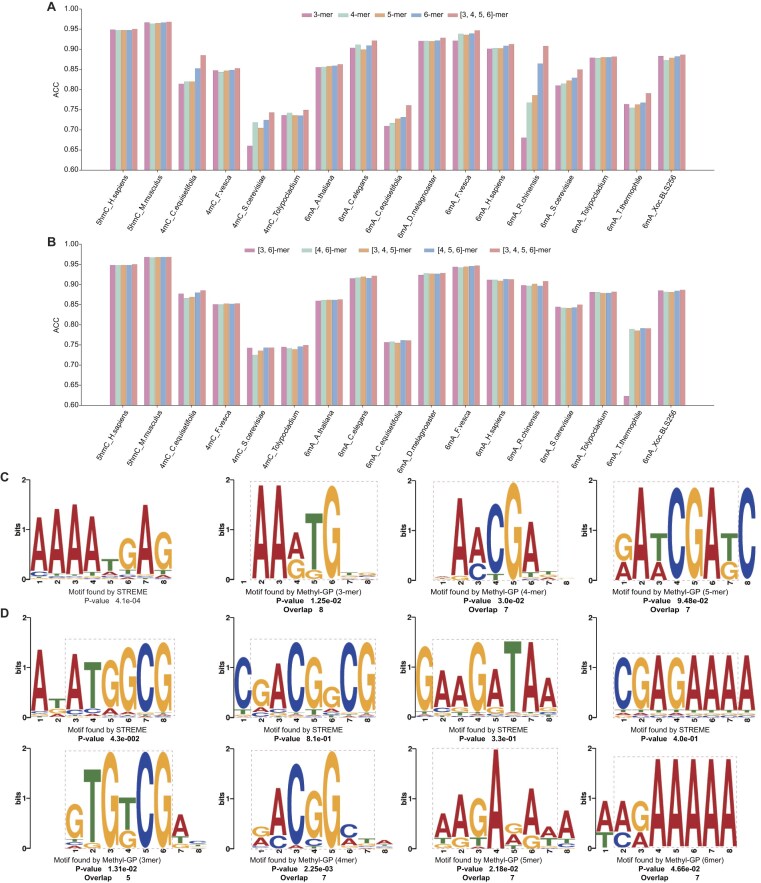
Performance of different k-mer combinations. (**A**) and (**B**) The ACC values of using single *k*-mer algorithms and combinations of k-mer algorithms to tokenize sequences on benchmark datasets, respectively. (**C**) The common motif of methylated sequences from the testing set of *6mA_A.thaliana* identified by STREME and Methyl-GP with different *k*-mer algorithms. (**D**) The unique motifs of methylated sequences from the testing set of *6mA_A.thaliana* identified by STREME and Methyl-GP. We set the length of the motifs identified by STREME to 8, and the *P*-value threshold to 0.5. For each aligned result, we first present the motifs discovered by STREME, with *P*-values calculated using the Fisher Exact Test. We then present motifs found by Methyl-GP, with the *P*-value and number of overlaps provided. The *P*-value is calculated by TOMTOM and represents the similarity between two motifs.

We used four *k*-mer algorithms to tokenize DNA sequences. To elucidate the impact of different *k*-mer combinations on model performance, we conducted ablation studies. We first compared the predictive ACC of various *k*-mer combinations. It can be observed that tokenizing DNA sequences using a single *k*-mer algorithm has advantages on different datasets, yet no consistent results are observed across all datasets (Fig. 6A). The average ACC achieved by using four *k*-mer algorithms simultaneously is significantly higher than that achieved by using a single *k*-mer algorithm, with margins of 3.51%, 2.54%, 2.39%, and 1.36%, respectively (Supplementary Table S6). We further compared the predictive ACC of different *k*-mer combinations (Fig. 6B). As we can see, the combination of four *k*-mer algorithms does not show a significant advantage. However, on average, it still achieves the highest predictive ACC. Specifically, the average ACC is 2.87% and 0.26% higher than that of the lowest predictive ACC combination ([3, 4]-mer) and the second highest combination ([3, 4, 6]-mer), respectively.

Then, to more intuitively illustrate the advantages of using four *k*-mers algorithms, we calculated attention scores to extract and visualize the corresponding motifs. Taking methylated sequences from the testing set of *6mA_A.thaliana* as an example, we presented the important motifs of these sequences based on STREME [46] and the attention mechanism, respectively. STREME is used to identify motifs that are enriched in the sequences. We compared the motifs found by both STREME and Methyl-GP. From (Fig. 6C), we can see that the motifs identified by STREME can also be discovered by Methyl-GP under different *k*-mer algorithms. This means that the sequence patterns corresponding to these motifs are very important for methylation identification. Meanwhile, Methyl-GP can also discover unique motifs that are identified only by specific *k*-mer algorithms (Fig. 6D). For example, the motif [G/A][A/C]CGGCG, which was found by STREME, is only discovered by Methyl-GP (4-mer). This suggests that some motifs are more easily found using specific *k*-mer algorithms. To further quantify the similarity between two motifs, we used TOMTOM [47] to calculate the statistical significance of motifs found by STREME and Methyl-GP, which is represented by the p-value. The lower the p-value, the more similar the two motifs appear to be. By comparing these motifs, it is evident that using the combination of four *k*-mer algorithms is more conducive to fully exploring sequence patterns, thereby enhancing the generalizability of the model to identify more important patterns related to DNA methylation. In other words, by combining different *k*-mer algorithms, the model can identify methylation regions more comprehensively, thereby avoiding the potential bias associated with tokenizing sequences using a single *k*-mer algorithm.

### Proposed fusion module generates higher-quality comprehensive representation

**Figure 7.**
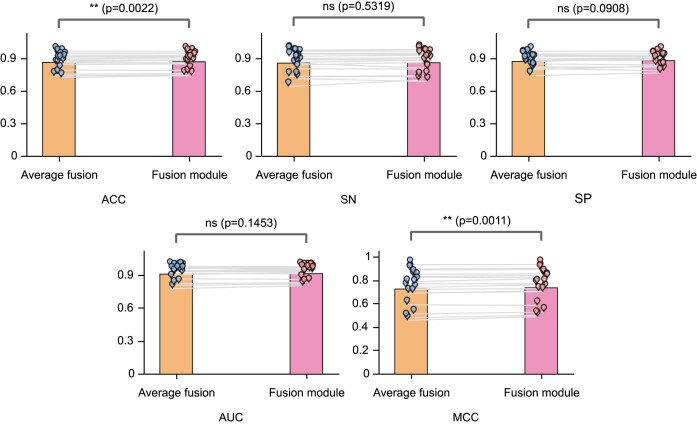
Ablation study of the proposed fusion module. Comparison of ACC, SN, SP, AUC, and MCC between the model with average fusion and the model with the proposed fusion module. A paired t-test was used to calculate the significant differences between these two methods, with ns indicates no significant difference, * represents *P* < 0.05, ** represents *P* < 0.01, and ***represents *P* < 0.001. The height of the bars represents the average value of the data.

In our study, we proposed a fusion module to integrate multiple embeddings extracted by the encoding module. To validate the effectiveness of our proposed fusion module, we compared it with the strategy of directly averaging the four embeddings. We used a paired t-test to determine whether there is a significant difference in the means of all results between the fusion module and average fusion strategy. As shown in (Fig. 7), there is a significant difference between the predictive ACC and MCC values of the fusion module and average fusion. This indicates that our method indeed improves the predictive performance of the model. The predictive ACC values of the fusion module are higher than those of average fusion on 15 out of 17 datasets, except for *6mA_Tolypocladium* and *6mA_T.thermophile* (Supplementary Table S7). It is also noteworthy that the performance improvement of the model on small datasets is significant after the introduction of the fusion module. Specifically, the ACC of the model with the fusion module on *4mC_C.equisetifolia*, *4mC_S.cerevisiae*, *4mC_Tolypocladium*, and *6mA_R.chinensis* is 1.63%, 1.82%, 1.02%, and 1.34% higher than that of the model with average fusion, respectively. The results demonstrate the effectiveness of the fusion module, particularly on small datasets, and further improve the generalizability of the model.

## Discussion

In this study, we proposed Methyl-GP for the identification of three types of DNA methylation. We compared our model with other state-of-the-art models on 17 benchmark datasets, and the results show that Methyl-GP is a powerful predictor. We also explored the influence of sequence patterns on methylation prediction. Through motif logo analysis and cross-species validation experiments, we observed that transfer learning between species with similar sequence patterns yields good results, indicating that the model is able to acquire generalizable knowledge. Conversely, transfer learning between species with significantly different sequence patterns resulted in unsatisfactory accuracies. This finding demonstrates that sequence patterns indeed play a significant role in methylation prediction. Additionally, we constructed phylogenetic trees to further analyze the relationship between evolutionary levels and sequence patterns among different species. This analysis revealed that evolutionary relationships may significantly influence the expression of methylation patterns. Recently, an increasing number of researchers have begun to leverage evolutionary relationships to address biological problems [48–50]. We believe that further exploration of the influence of evolutionary relationships on methylation patterns could greatly aid in methylation identification and represents a promising direction for future research.

We also attempted to extract and visualize motifs corresponding to important regions of sequences by calculating attention scores for tokens. To quantify the importance of these motifs, we used STREME to identify motifs within sequences and compared them with those discovered by our model. Next, we used TOMTOM to calculate the similarity between the two sets of motifs. This comparison across several sets of motifs suggests that our model can adaptively capture important sequence regions.

Although current methylation identification methods can achieve satisfactory results relying solely on sequence-based input, there remain multiple directions to explore in future research. Firstly, augmenting the input modalities and features may further improve the predictive performance of the model. By integrating sequence-based features with other types of features, such as those derived from genomic data, the model is capable of making more accurate decisions. [51–53]. Furthermore, electrical signals data generated by third-generation sequencing methods, such as nanopore sequencing technology, can serve as an additional input to guide the learning process of the model. Secondly, as mentioned above, thoroughly exploring the evolutionary relationships among different species and extracting latent information can assist in constructing more rational models, thereby aiding the identification of methylation. Thirdly, while our analysis focuses on local sequence regions surrounding methylation sites, long-range interactive effects of gene regulation in the genome, such as enhance-promoter interactions, may also play a significant role [54]. Therefore, it is also crucial to explore how long-range sequence integrative information influences DNA methylation levels. Finally, identification methods based on single-nucleotide resolution may enable us to broaden our perspective and explore a wider range of methylation patterns.

## Supplementary Material

gkaf223_Supplemental_File

## Data Availability

The benchmark datasets used for comparing model performance are derived from the study by Lv et al. and are available at https://doi.org/10.1016/j.isci.2020.100991. All original code of this study has been deposited at GitHub at https://github.com/Hao010418/Mehtyl-GP and Zenodo at https://doi.org/10.5281/zenodo.14207713.
