# Supplementary material for: Methyl-GP: accurate generic DNA methylation prediction based on a language model and representation learning
Source: Nucleic Acids Res. 2025 Mar 28;53(6):gkaf223. doi: 10.1093/nar/gkaf223 (PMC11952970; doi:10.1093/nar/gkaf223)
Supplement: gkaf223_Supplemental_File [file gkaf223_supplemental_file.pdf]

Table S1. The details of 17 benchmark datasets.

Species	4mC				5hmC				6mA			
	Training set		Testing set		Training set		Testing set		Training set		Testing set	
	pos	neg	pos	neg	pos	neg	pos	neg	pos	neg	pos	neg
A.thaliana	-	-	-	-	-	-	-	-	15937	15937	15936	15936
C.elegans	-	-	-	-	-	-	-	-	3981	3981	3980	3980
C.equisetifolia	183	183	183	183	-	-	-	-	3033	3033	3033	3033
D.melanogaster	-	-	-	-	-	-	-	-	5596	5596	5595	5595
F.vesca	7899	7899	7898	7898	-	-	-	-	1551	1551	1551	1551
H.sapiens	-	-	-	-	1172	1172	1172	1172	9168	9168	9167	9167
M.musculus	-	-	-	-	1840	1840	1839	1839	-	-	-	-
R.chinensis	-	-	-	-	-	-	-	-	300	300	299	299
S.cerevisiae	990	990	989	989	-	-	-	-	1893	1893	1893	1893
Tolypocladium	7664	7664	7663	7663	-	-	-	-	53800	53800	53800	53800
T.thermophila	-	-	-	-	-	-	-	-	1690	1690	1689	1689
Xoc.BLS256	-	-	-	-	-	-	-	-	8608	8608	8607	8607

Table S2. The training parameters of Methyl-GP on 17 benchmark datasets.

Methylation Type	Species	Batch Size	Learning rate	Weight decay	Pretrain_epoch
**5hmC**	H.sapiens	128	0.00005	0.003	20
	M.musculus	128	0.00005	0.003	20
**4mC**	C.equisetifolia	64	0.00005	0.003	20
	F.vesca	64	0.00003	0.001	20
	S.cerevisiae	128	0.00005	0.003	20
	Tolypocladium	128	0.00003	0.001	20
**6mA**	A.thaliana	128	0.00005	0.003	10
	C.elegans	64	0.00005	0.003	20
	C.equisetifolia	128	0.00005	0.003	20
	D.melanogaster	128	0.00005	0.003	20
	F.vesca	128	0.00005	0.003	20
	H.sapiens	128	0.00005	0.003	20
	R.chinensis	64	0.00005	0.003	20
	S.cerevisiae	128	0.00005	0.003	20
	T.thermophila	128	0.00005	0.003	5
	Tolypocladium	128	0.00005	0.003	20
	Xoc.BLS256	128	0.00005	0.003	20

Table S3. Performances of Methyl-GP and other state-of-the-art predictors on 17 benchmark datasets.

Methylation Type	Species	Model	ACC	SN	SP	AUC	MCC
**5hmC**	H.sapiens	iDNA-MS	0.9475	0.9770	0.9181	0.9600	0.8970
		Deep6mA	0.9445	0.9761	0.9130	0.9656	0.8909
		Bert6mA	0.9454	0.9753	0.9155	0.9565	0.8924
		iDNA-ABF	0.9501	0.9838	0.9164	0.9677	0.9022
		Methyl-GP	0.9501	0.9829	0.9172	0.9579	0.9021
	M.musculus	iDNA-MS	0.9679	0.9685	0.9668	0.9840	0.9360
		Deep6mA	0.9655	0.9695	0.9614	0.9777	0.9310
		Bert6mA	0.9622	0.9690	0.9554	0.9813	0.9245
		iDNA-ABF	0.9679	0.9690	0.9668	0.9796	0.9358
		Methyl-GP	0.9679	0.9690	0.9668	0.9827	0.9358
**4mC**	C.equisetifolia	iDNA-MS	0.7109	0.7169	0.7049	0.7800	0.4220
		Deep6mA	0.8169	0.7049	0.9290	0.8902	0.6504
		Bert6mA	0.7486	0.7049	0.7923	0.8204	0.4992
		iDNA-ABF	0.8579	0.8743	0.8415	0.9089	0.7162
		Methyl-GP	0.8852	0.8634	0.9071	0.9308	0.7600
	F.vesca	iDNA-MS	0.8239	0.8297	0.8181	0.9000	0.6480
		Deep6mA	0.8449	0.8703	0.8194	0.9194	0.6907
		Bert6mA	0.8317	0.8373	0.8262	0.9103	0.6635
		iDNA-ABF	0.8524	0.8535	0.8512	0.9285	0.7047
		Methyl-GP	0.8528	0.8847	0.8210	0.9235	0.7071
	S.cerevisiae	iDNA-MS	0.7042	0.7017	0.7068	0.7710	0.4080
		Deep6mA	0.7053	0.7240	0.6866	0.7735	0.4108
		Bert6mA	0.7184	0.6785	0.7583	0.7820	0.4382
		iDNA-ABF	0.7230	0.6876	0.7583	0.7897	0.4470
		Methyl-GP	0.7432	0.6997	0.7867	0.8075	0.4882
	Tolypocladium	iDNA-MS	0.7115	0.7159	0.7076	0.7800	0.4230
		Deep6mA	0.7310	0.7374	0.7247	0.8059	0.4621
		Bert6mA	0.7277	0.7308	0.7247	0.8006	0.4554
		iDNA-ABF	0.7434	0.7385	0.7483	0.8213	0.4868
		Methyl-GP	0.7494	0.7316	0.7672	0.8241	0.4991
**6mA**	A.thaliana	iDNA-MS	0.8377	0.8244	0.8511	0.9110	0.6760
		Deep6mA	0.8624	0.8412	0.8835	0.9341	0.7254
		Bert6mA	0.8557	0.8383	0.8732	0.9277	0.7120
		iDNA-ABF	0.8603	0.8264	0.8942	0.9349	0.7223
		Methyl-GP	0.8627	0.8107	0.9148	0.9379	0.7294
	C.elegans	iDNA-MS	0.8557	0.8676	0.8437	0.9350	0.7120
		Deep6mA	0.8967	0.9384	0.8550	0.9602	0.7962
		Bert6mA	0.8940	0.9013	0.8867	0.9577	0.7880
		iDNA-ABF	0.9138	0.9256	0.9020	0.9682	0.8279
		Methyl-GP	0.9214	0.9384	0.9043	0.9727	0.8432
	C.equisetifolia	iDNA-MS	0.7113	0.7181	0.7046	0.7790	0.4230
		Deep6mA	0.7221	0.7343	0.7099	0.7957	0.4442
		Bert6mA	0.7219	0.6805	0.7633	0.7923	0.4453
		iDNA-ABF	0.7399	0.6713	0.8084	0.8098	0.4843
		Methyl-GP	0.7610	0.6894	0.8325	0.8358	0.5274
	D.melanogaster	iDNA-MS	0.8962	0.8897	0.9026	0.9560	0.7920
		Deep6mA	0.9164	0.9237	0.9090	0.9660	0.8328
		Bert6mA	0.9148	0.9108	0.9189	0.9658	0.8297
		iDNA-ABF	0.9228	0.9301	0.9155	0.9713	0.8457
		Methyl-GP	0.9283	0.9301	0.9265	0.9740	0.8567
	F.vesca	iDNA-MS	0.9226	0.9394	0.9226	0.9770	0.8460
		Deep6mA	0.9290	0.9375	0.9207	0.9738	0.8583
		Bert6mA	0.9191	0.9201	0.9181	0.9732	0.8382
		iDNA-ABF	0.9413	0.9452	0.9375	0.9804	0.8827
		Methyl-GP	0.9468	0.9491	0.9446	0.9711	0.8936
	H.sapiens	iDNA-MS	0.8842	0.8631	0.9052	0.9500	0.7690
		Deep6mA	0.9060	0.8965	0.9156	0.9661	0.8122
		Bert6mA	0.8975	0.8900	0.9049	0.9628	0.7950
		iDNA-ABF	0.9104	0.9057	0.9151	0.9695	0.8209
		Methyl-GP	0.9126	0.9038	0.9215	0.9718	0.8254
	R.chinensis	iDNA-MS	0.8545	0.8796	0.8294	0.9240	0.7100
		Deep6mA	0.8177	0.8294	0.8060	0.9023	0.6356
		Bert6mA	0.7826	0.7860	0.7793	0.8420	0.5652

		iDNA-ABF	0.8629	0.8328	0.8930	0.9280	0.7271
		Methyl-GP	0.9080	0.9097	0.9064	0.9595	0.8161
	S.cerevisiae	iDNA-MS	0.7855	0.7538	0.8172	0.8680	0.5720
		Deep6mA	0.8035	0.8024	0.8045	0.8870	0.6070
		Bert6mA	0.8172	0.7913	0.8431	0.8925	0.6353
		iDNA-ABF	0.8278	0.7966	0.8590	0.9062	0.6569
		Methyl-GP	0.8497	0.8024	0.8970	0.9180	0.7026
	T.thermophila	iDNA-MS	0.8563	0.9579	0.7548	0.9220	0.7280
		Deep6mA	0.8664	0.9427	0.7902	0.9271	0.7415
		Bert6mA	0.8756	0.9292	0.8220	0.9389	0.7555
		iDNA-ABF	0.8804	0.9442	0.8167	0.9355	0.7671
		Methyl-GP	0.8818	0.9422	0.8214	0.9442	0.7692
	Tolypocladium	iDNA-MS	0.7342	0.7425	0.7259	0.8130	0.4680
		Deep6mA	0.7425	0.8259	0.6590	0.8181	0.4918
		Bert6mA	0.7427	0.7306	0.7549	0.8238	0.4856
		iDNA-ABF	0.7771	0.7649	0.7892	0.8500	0.5543
		Methyl-GP	0.7910	0.7413	0.8407	0.8660	0.5849
	Xoc.BLS256	iDNA-MS	0.8451	0.8250	0.8652	0.9210	0.6910
		Deep6mA	0.8547	0.8851	0.8242	0.9307	0.7106
		Bert6mA	0.8639	0.8527	0.8751	0.9341	0.7280
		iDNA-ABF	0.8817	0.8808	0.8827	0.9506	0.7634
		Methyl-GP	0.8864	0.8883	0.8844	0.9520	0.7727

Table S4. Predictive ACC of the cross-species validation on benchmark datasets.

Test \ Train	6mAF	6mAR	6mACEQ	6mAA	6mAT	6mAS	6mACEL	6mAD	6mAH	6mATT	6mAX
6mAF	0.9468	0.9113	0.8926	0.9217	0.8565	0.8907	0.7979	0.9094	0.9046	0.7817	0.7563
6mAR	0.9097	0.9080	0.8679	0.8846	0.8495	0.8579	0.7809	0.8612	0.8696	0.7642	0.7425
6mACEQ	0.7166	0.7257	0.7610	0.7549	0.7372	0.7356	0.7128	0.7181	0.7427	0.5706	0.6601
6mAA	0.8362	0.8410	0.8476	0.8627	0.8221	0.8453	0.8066	0.8401	0.8514	0.5790	0.7186
6mAT	0.7250	0.7558	0.7647	0.7496	0.8818	0.7522	0.7087	0.7244	0.7433	0.5817	0.6495
6mAS	0.7752	0.7953	0.8104	0.8032	0.8027	0.8497	0.8077	0.8061	0.7993	0.5637	0.7472
6mACEL	0.6843	0.7216	0.8427	0.7829	0.8344	0.8207	0.9214	0.7340	0.8046	0.5523	0.6948
6mAD	0.9009	0.8979	0.8782	0.9136	0.8590	0.8958	0.8669	0.9283	0.8928	0.6420	0.8363
6mAH	0.8808	0.8794	0.8881	0.8936	0.8675	0.8768	0.8616	0.8792	0.9126	0.6060	0.7670
6mATT	0.5414	0.5846	0.6053	0.5878	0.5692	0.5590	0.5566	0.5459	0.5647	0.7910	0.5027
6mAX	0.6831	0.7331	0.8213	0.7640	0.7832	0.7605	0.7421	0.7241	0.7189	0.4503	0.8864

Test \ Train	4mCC	4mCF	4mCS	4mCT
4mCC	0.8852	0.7923	0.7322	0.7514
4mCF	0.8077	0.8528	0.7666	0.7937
4mCS	0.6638	0.6582	0.7432	0.6962
4mCT	0.6865	0.6971	0.6957	0.7494

Test \ Train	5hmCH	5hmCM
5hmCH	0.9501	0.9475
5hmCM	0.9676	0.9679

Table S5. Performance of different fine-tuning strategies on 17 benchmark datasets.

Methylation Type	Species	Strategies	ACC	SN	SP	AUC	MCC
**5hmC**	H.sapiens	Methyl-GP-NF	0.9488	0.9812	0.9164	0.9570	0.8995
		Methyl-GP-FS	0.9501	0.9838	0.9164	0.9686	0.9022
		Methyl-GP	0.9501	0.9829	0.9172	0.9579	0.9021
	M.musculus	Methyl-GP-NF	0.9676	0.9685	0.9668	0.9866	0.9353
		Methyl-GP-FS	0.9679	0.9690	0.9668	0.9872	0.9358
		Methyl-GP	0.9679	0.9690	0.9668	0.9827	0.9358
**4mC**	C.equisetifolia	Methyl-GP-NF	0.8525	0.8306	0.8743	0.9082	0.7056
		Methyl-GP-FS	0.8634	0.8962	0.8306	0.9117	0.7283
		Methyl-GP	0.8852	0.8634	0.9071	0.9308	0.7600
	F.vesca	Methyl-GP-NF	0.8520	0.8947	0.8093	0.9247	0.7066
		Methyl-GP-FS	0.8514	0.8749	0.8278	0.9257	0.7035
		Methyl-GP	0.8528	0.8847	0.8210	0.9235	0.7071
	S.cerevisiae	Methyl-GP-NF	0.7285	0.6815	0.7755	0.7847	0.4591
		Methyl-GP-FS	0.7366	0.6623	0.8109	0.7829	0.4785
		Methyl-GP	0.7432	0.6997	0.7867	0.8075	0.4882
	Tolypocladium	Methyl-GP-NF	0.7325	0.7802	0.6848	0.8121	0.4672
		Methyl-GP-FS	0.7504	0.7612	0.7397	0.8266	0.5010
		Methyl-GP	0.7494	0.7316	0.7672	0.8241	0.4991
**6mA**	A.thaliana	Methyl-GP-NF	0.8599	0.8379	0.8820	0.9346	0.7206
		Methyl-GP-FS	0.8608	0.8353	0.8862	0.9345	0.7225
		Methyl-GP	0.8627	0.8107	0.9148	0.9379	0.7294
	C.elegans	Methyl-GP-NF	0.9122	0.9060	0.9183	0.9679	0.8244
		Methyl-GP-FS	0.9149	0.9188	0.9111	0.9657	0.8299
		Methyl-GP	0.9214	0.9384	0.9043	0.9727	0.8432
	C.equisetifolia	Methyl-GP-NF	0.7417	0.6888	0.7946	0.8161	0.4861
		Methyl-GP-FS	0.7390	0.6904	0.7877	0.8104	0.4804
		Methyl-GP	0.7610	0.6894	0.8325	0.8358	0.5274
	D.melanogaster	Methyl-GP-NF	0.9220	0.9219	0.9221	0.9683	0.8440
		Methyl-GP-FS	0.9230	0.9301	0.9158	0.9657	0.8460
		Methyl-GP	0.9283	0.9301	0.9265	0.9740	0.8567
	F.vesca	Methyl-GP-NF	0.9400	0.9413	0.9387	0.9779	0.8801
		Methyl-GP-FS	0.9420	0.9349	0.9491	0.9752	0.8840
		Methyl-GP	0.9468	0.9491	0.9446	0.9711	0.8936
	H.sapiens	Methyl-GP-NF	0.9097	0.8956	0.9237	0.9695	0.8197
		Methyl-GP-FS	0.9103	0.9039	0.9168	0.9668	0.8207
		Methyl-GP	0.9126	0.9038	0.9215	0.9718	0.8254
	R.chinensis	Methyl-GP-NF	0.8378	0.8495	0.8261	0.9009	0.6758
		Methyl-GP-FS	0.8779	0.8763	0.8796	0.9467	0.7559
		Methyl-GP	0.9080	0.9097	0.9064	0.9595	0.8161
	S.cerevisiae	Methyl-GP-NF	0.8114	0.7771	0.8457	0.8767	0.6243
		Methyl-GP-FS	0.8299	0.8236	0.8362	0.8958	0.6599
		Methyl-GP	0.8497	0.8024	0.8970	0.9180	0.7026
	Tolypocladium	Methyl-GP-NF	0.7703	0.7880	0.7525	0.8523	0.5409
		Methyl-GP-FS	0.7795	0.7555	0.8034	0.8488	0.5596
		Methyl-GP	0.7910	0.7413	0.8407	0.8660	0.5849
	T.thermophila	Methyl-GP-NF	0.8808	0.9516	0.8101	0.9419	0.7694
		Methyl-GP-FS	0.8811	0.9323	0.8298	0.9442	0.7661
		Methyl-GP	0.8818	0.9422	0.8214	0.9442	0.7692
	Xoc.BLS256	Methyl-GP-NF	0.6647	0.4229	0.9066	0.7708	0.3765
		Methyl-GP-FS	0.8842	0.8775	0.8911	0.9467	0.7684
		Methyl-GP	0.8864	0.8883	0.8844	0.9520	0.7727

Table S6. Performance of different k-mer combinations.

Methylation Type	Species	Combination	ACC	SN	SP	AUC	MCC
**5hmC**	H.sapiens	3-mer	0.9488	0.9488	0.9488	0.9690	0.8994
		4-mer	0.9471	0.9471	0.9471	0.9708	0.8958
		5-mer	0.9475	0.9475	0.9475	0.9671	0.8966
		6-mer	0.9475	0.9475	0.9475	0.9686	0.8970
		[3, 4]-mer	0.9471	0.9812	0.9130	0.9693	0.8963
		[3, 5]-mer	0.9484	0.9787	0.9181	0.9685	0.8984
		[3, 6]-mer	0.9480	0.9778	0.9181	0.9728	0.8975
		[4, 5]-mer	0.9475	0.9770	0.9181	0.9665	0.8966
		[4, 6]-mer	0.9509	0.9863	0.9155	0.9564	0.9041
		[5, 6]-mer	0.9484	0.9770	0.9198	0.9606	0.8982
		[3, 4, 5]-mer	0.9480	0.9778	0.9181	0.9643	0.8975
		[3, 4, 6]-mer	0.9480	0.9787	0.9172	0.9621	0.8976
		[3, 5, 6]-mer	0.9475	0.9770	0.9181	0.9710	0.8966
		[4, 5, 6]-mer	0.9505	0.9855	0.9155	0.9649	0.9032
		[3, 4, 5, 6]-mer	0.9501	0.9829	0.9172	0.9579	0.9021
	M.musculus	3-mer	0.9666	0.9666	0.9666	0.9806	0.9331
		4-mer	0.9633	0.9633	0.9633	0.9801	0.9266
		5-mer	0.9649	0.9649	0.9649	0.9805	0.9299
		6-mer	0.9663	0.9663	0.9663	0.9870	0.9326
		[3, 4]-mer	0.9674	0.9685	0.9663	0.9822	0.9347
		[3, 5]-mer	0.9674	0.9690	0.9657	0.9835	0.9348
		[3, 6]-mer	0.9679	0.9690	0.9668	0.9852	0.9358
		[4, 5]-mer	0.9668	0.9695	0.9641	0.9839	0.9337
		[4, 6]-mer	0.9676	0.9685	0.9668	0.9841	0.9353
		[5, 6]-mer	0.9676	0.9690	0.9663	0.9847	0.9353
		[3, 4, 5]-mer	0.9676	0.9690	0.9663	0.9821	0.9353
		[3, 4, 6]-mer	0.9679	0.9690	0.6668	0.9832	0.9358
		[3, 5, 6]-mer	0.9676	0.9690	0.9663	0.9835	0.9353
		[4, 5, 6]-mer	0.9676	0.9685	0.9668	0.9842	0.9353
		[3, 4, 5, 6]-mer	0.9679	0.9690	0.9668	0.9827	0.9358
**4mC**	C.equisetifolia	3-mer	0.8142	0.8142	0.8142	0.8871	0.6330
		4-mer	0.8197	0.8197	0.8197	0.8746	0.6400
		5-mer	0.8197	0.8197	0.8197	0.8735	0.6407
		6-mer	0.8525	0.8525	0.8525	0.9059	0.7064
		[3, 4]-mer	0.8497	0.8251	0.8743	0.9095	0.7004
		[3, 5]-mer	0.8607	0.8579	0.8634	0.9285	0.7213
		[3, 6]-mer	0.8770	0.8689	0.8852	0.9176	0.7542
		[4, 5]-mer	0.8661	0.8634	0.8689	0.9235	0.7323
		[4, 6]-mer	0.8689	0.8689	0.8689	0.9239	0.7377
		[5, 6]-mer	0.8716	0.8579	0.8852	0.9177	0.7434
		[3, 4, 5]-mer	0.8689	0.8361	0.9016	0.9187	0.7393
		[3, 4, 6]-mer	0.8798	0.8525	0.9071	0.9201	0.7607
		[3, 5, 6]-mer	0.8743	0.8306	0.9180	0.9205	0.7515
		[4, 5, 6]-mer	0.8825	0.8743	0.8907	0.9168	0.7651
		[3, 4, 5, 6]-mer	0.8852	0.8634	0.9071	0.9197	0.7712
	F.vesca	3-mer	0.8476	0.8476	0.8476	0.9158	0.6958
		4-mer	0.8437	0.8437	0.8437	0.9189	0.6882
		5-mer	0.8469	0.8469	0.8469	0.9193	0.6938
		6-mer	0.8484	0.8484	0.8484	0.9257	0.6971
		[3, 4]-mer	0.8398	0.8470	0.8325	0.9039	0.6796
		[3, 5]-mer	0.8489	0.8633	0.8346	0.9238	0.6982
		[3, 6]-mer	0.8507	0.8510	0.8505	0.9265	0.7014
		[4, 5]-mer	0.8503	0.8745	0.8260	0.9252	0.7014
		[4, 6]-mer	0.8493	0.8734	0.8251	0.9238	0.6993
		[5, 6]-mer	0.8505	0.8593	0.8416	0.9266	0.7010
		[3, 4, 5]-mer	0.8524	0.8816	0.8232	0.9246	0.7061
		[3, 4, 6]-mer	0.8517	0.8527	0.8506	0.9277	0.7033
		[3, 5, 6]-mer	0.8510	0.8821	0.8198	0.9031	0.7033
		[4, 5, 6]-mer	0.8526	0.8748	0.8305	0.9271	0.7059
		[3, 4, 5, 6]-mer	0.8528	0.8847	0.8210	0.9235	0.7071
	S.cerevisiae	3-mer	0.6603	0.6603	0.6603	0.7384	0.3309
		4-mer	0.7184	0.7184	0.7184	0.7765	0.4379
		5-mer	0.7048	0.7048	0.7048	0.7714	0.4165

		6-mer	0.7245	0.7245	0.7245	0.7862	0.7243
		[3, 4]-mer	0.7128	0.7108	0.7149	0.7579	0.4257
		[3, 5]-mer	0.7341	0.6775	0.7907	0.7970	0.4712
		[3, 6]-mer	0.7427	0.6845	0.8008	0.8092	0.4887
		[4, 5]-mer	0.7250	0.6320	0.8180	0.7954	0.4579
		[4, 6]-mer	0.7452	0.7159	0.7745	0.8034	0.4912
		[5, 6]-mer	0.7432	0.7118	0.7745	0.7938	0.4873
		[3, 4, 5]-mer	0.7356	0.7108	0.7604	0.8015	0.4718
		[3, 4, 6]-mer	0.7432	0.6946	0.7917	0.8031	0.4887
		[3, 5, 6]-mer	0.7356	0.6431	0.8281	0.7866	0.4795
		[4, 5, 6]-mer	0.7326	0.6977	0.7674	0.7937	0.4663
		[3, 4, 5, 6]-mer	0.7432	0.6997	0.7867	0.8075	0.4882
	Tolypocladium	3-mer	0.7363	0.7363	0.7363	0.8140	0.4749
		4-mer	0.7421	0.7421	0.7421	0.8194	0.4852
		5-mer	0.7361	0.7361	0.7361	0.8139	0.4723
		6-mer	0.7354	0.7354	0.7354	0.8119	0.4719
		[3, 4]-mer	0.6437	0.4404	0.8471	0.7251	0.3147
		[3, 5]-mer	0.7410	0.7399	0.7420	0.8145	0.4819
		[3, 6]-mer	0.7450	0.6876	0.8024	0.8233	0.4933
		[4, 5]-mer	0.7414	0.7399	0.7428	0.8160	0.4827
		[4, 6]-mer	0.7436	0.7885	0.6988	0.8219	0.4892
		[5, 6]-mer	0.7444	0.7625	0.7262	0.8207	0.4890
		[3, 4, 5]-mer	0.7391	0.7712	0.7069	0.8162	0.4791
		[3, 4, 6]-mer	0.7459	0.7500	0.7419	0.8228	0.4919
		[3, 5, 6]-mer	0.7467	0.7504	0.7431	0.8210	0.4934
		[4, 5, 6]-mer	0.7468	0.7856	0.7079	0.8225	0.4950
		[3, 4, 5, 6]-mer	0.7494	0.7316	0.7672	0.8241	0.4991
**6mA**	A.thaliana	3-mer	0.8553	0.8553	0.8553	0.9308	0.7105
		4-mer	0.8560	0.8560	0.8560	0.9296	0.7121
		5-mer	0.8580	0.8580	0.8580	0.9325	0.7165
		6-mer	0.8591	0.8591	0.8591	0.9326	0.7185
		[3, 4]-mer	0.8499	0.8349	0.8650	0.9239	0.7002
		[3, 5]-mer	0.8573	0.8605	08540	0.9331	0.7146
		[3, 6]-mer	0.8594	0.8663	0.8525	0.9367	0.7189
		[4, 5]-mer	0.8609	0.8295	0.8923	0.9342	0.7233
		[4, 6]-mer	0.8590	0.8729	0.8451	0.9353	0.7183
		[5, 6]-mer	0.8589	0.8808	0.8369	0.9369	0.7184
		[3, 4, 5]-mer	0.8617	0.8127	0.9108	0.9360	0.7270
		[3, 4, 6]-mer	0.8614	0.8498	0.8731	0.9352	0.7231
		[3, 5, 6]-mer	0.8630	0.8562	0.8699	0.9373	0.7262
		[4, 5, 6]-mer	0.8637	0.8197	0.9078	0.9374	0.7302
		[3, 4, 5, 6]-mer	0.8627	0.8107	0.9148	0.9379	0.7294
	C.elegans	3-mer	0.9034	0.9034	0.9034	0.9608	0.8074
		4-mer	0.9112	0.9112	0.9112	0.9572	0.8226
		5-mer	0.8994	0.8994	0.8994	0.9616	0.7988
		6-mer	0.9093	0.9093	0.9093	0.9665	0.8194
		[3, 4]-mer	0.9072	0.9344	0.8799	0.9629	0.8155
		[3, 5]-mer	0.9153	0.9294	0.9013	0.9685	0.8310
		[3, 6]-mer	0.9151	0.9276	0.9025	0.9631	0.8304
		[4, 5]-mer	0.9167	0.9364	0.8970	0.9654	0.8341
		[4, 6]-mer	0.9155	0.9374	0.8935	0.9652	0.8317
		[5, 6]-mer	0.9176	0.9269	0.9083	0.9645	0.8353
		[3, 4, 5]-mer	0.9192	0.9352	0.9033	0.9714	0.8389
		[3, 4, 6]-mer	0.9156	0.9337	0.8975	0.9662	0.8317
		[3, 5, 6]-mer	0.9183	0.9146	0.9221	0.9715	0.8367
		[4, 5, 6]-mer	0.9157	0.9201	0.9113	0.9709	0.8314
		[3, 4, 5, 6]-mer	0.9214	0.9384	0.9043	0.9727	0.8432
	C.equisetifolia	3-mer	0.7094	0.7094	0.7094	0.7850	0.4193
		4-mer	0.7169	0.7169	0.7169	0.7892	0.4361
		5-mer	0.7277	0.7277	0.7277	0.8044	0.4566
		6-mer	0.7316	0.7316	0.7316	0.8049	0.4655
		[3, 4]-mer	0.7468	0.6601	0.8335	0.8211	0.5012
		[3, 5]-mer	0.7587	0.7336	0.7837	0.8317	0.5180
		[3, 6]-mer	0.7567	0.6673	0.8460	0.8336	0.5218

		[4, 5]-mer	0.7578	0.7303	0.7854	0.8281	0.5164
		[4, 6]-mer	0.7595	0.6927	0.8262	0.8309	0.5236
		[5, 6]-mer	0.7575	0.6947	0.8231	0.8329	0.5191
		[3, 4, 5]-mer	0.7550	0.7524	0.7577	0.8297	0.5101
		[3, 4, 6]-mer	0.7615	0.6973	0.8256	0.8351	0.5273
		[3, 5, 6]-mer	0.7577	0.6911	0.8243	0.8337	0.5200
		[4, 5, 6]-mer	0.7565	0.7039	0.8091	0.8321	0.5159
		[3, 4, 5, 6]-mer	0.7610	0.6894	0.8325	0.8358	0.5274
	D.melanogaster	3-mer	0.9206	0.9206	0.9206	0.9619	0.8413
		4-mer	0.9187	0.9187	0.9187	0.9623	0.8374
		5-mer	0.9200	0.9200	0.9200	0.9662	0.8401
		6-mer	0.9217	0.9217	0.9217	0.9657	0.8434
		[3, 4]-mer	0.9212	0.9262	0.9162	0.9681	0.8424
		[3, 5]-mer	0.9254	0.9251	0.9256	0.9734	0.8508
		[3, 6]-mer	0.9236	0.9240	0.9231	0..9627	0.8472
		[4, 5]-mer	0.9275	0.9237	0.9314	0.9736	0.8551
		[4, 6]-mer	0.9250	0.9292	0.9208	0.9687	0.8501
		[5, 6]-mer	0.9286	0.9253	0.9319	0.9743	0.8572
		[3, 4, 5]-mer	0.9266	0.9183	0.9349	0.9738	0.8534
		[3, 4, 6]-mer	0.9265	0.9378	0.9151	0.9751	0.8531
		[3, 5, 6]-mer	0.9285	0.9185	0.9385	0.9731	0.8572
		[4, 5, 6]-mer	0.9268	0.9260	0.9276	0.9744	0.8536
		[3, 4, 5, 6]-mer	0.9283	0.9301	0.9265	0.9740	0.8567
	F.vesca	3-mer	0.9213	0.9213	0.9213	0.9681	0.8427
		4-mer	0.9381	0.9381	0.9381	0.9781	0.8762
		5-mer	0.9358	0.9358	0.9358	0.9807	0.8718
		6-mer	0.9391	0.9391	0.9391	0.9804	0.8781
		[3, 4]-mer	0.9400	0.9439	0.9381	0.9820	0.8820
		[3, 5]-mer	0.9436	0.9426	0.9446	0.9747	0.8872
		[3, 6]-mer	0.9436	0.9587	0.9284	0.9837	0.8876
		[4, 5]-mer	0.9420	0.9407	0.9433	0.9844	0.8839
		[4, 6]-mer	0.9429	0.9516	0.9342	0.9812	0.8860
		[5, 6]-mer	0.9439	0.9607	0.9271	0.9835	0.8883
		[3, 4, 5]-mer	0.9442	0.9375	0.9510	0.9831	0.8885
		[3, 4, 6]-mer	0.9455	0.9465	0.9446	0.9726	0.8910
		[3, 5, 6]-mer	0.9458	0.9478	0.9439	0.9782	0.8917
		[4, 5, 6]-mer	0.9446	0.9536	0.9355	0.9696	0.8892
		[3, 4, 5, 6]-mer	0.9468	0.9491	0.9446	0.9711	0.8936
	H.sapiens	3-mer	0.9013	0.9013	0.9013	0.9648	0.8032
		4-mer	0.9029	0.9029	0.9029	0.9627	0.8057
		5-mer	0.9023	0.9023	0.9023	0.9540	0.8048
		6-mer	0.9085	0.9085	0.9085	0.9674	0.8170
		[3, 4]-mer	0.9030	0.9240	0.8820	0.9630	0.8066
		[3, 5]-mer	0.9104	0.9017	0.9192	0.9703	0.8210
		[3, 6]-mer	0.9114	0.8953	0.9275	0.9695	0.8232
		[4, 5]-mer	0.9114	0.9030	0.9198	0.9710	0.8830
		[4, 6]-mer	0.9109	0.8899	0.9319	0.9697	0.8226
		[5, 6]-mer	0.9122	0.8885	0.9359	0.9717	0.8253
		[3, 4, 5]-mer	0.9089	0.9011	0.9168	0.9623	0.8179
		[3, 4, 6]-mer	0.9131	0.9013	0.9249	0.9712	0.8265
		[3, 5, 6]-mer	0.9111	0.9067	0.9155	0.9616	0.8222
		[4, 5, 6]-mer	0.9152	0.9197	0.9107	0.9724	0.8304
		[3, 4, 5, 6]-mer	0.9126	0.9038	0.9215	0.9718	0.8254
	R.chinensis	3-mer	0.6806	0.6806	0.6806	0.7386	0.3634
		4-mer	0.7676	0.7676	0.7676	0.8476	0.5351
		5-mer	0.7860	0.7860	0.7860	0.8584	0.5727
		6-mer	0.8645	0.8645	0.8645	0.9384	0.7309
		[3, 4]-mer	0.7174	0.5452	0.8896	0.7981	0.4631
		[3, 5]-mer	0.8997	0.9097	0.8896	0.9517	0.7995
		[3, 6]-mer	0.8980	0.9130	0.8829	0.8564	0.7963
		[4, 5]-mer	0.8963	0.9030	0.8896	0.9468	0.7927
		[4, 6]-mer	0.8863	0.8829	0.8896	0.9427	0.7726
		[5, 6]-mer	0.8997	0.8997	0.8997	0.9546	0.7993
		[3, 4, 5]-mer	0.9013	0.9064	0.8963	0.9375	0.8027

		[3, 4, 6]-mer	0.8963	0.8829	0.9097	0.9527	0.7929
		[3, 5, 6]-mer	0.8980	0.9130	0.8829	0.9392	0.7963
		[4, 5, 6]-mer	0.8946	0.8896	0.8997	0.9531	0.7893
		[3, 4, 5, 6]-mer	0.9080	0.9097	0.9064	0.9595	0.8161
	S.cerevisiae	3-mer	0.8101	0.8101	0.8101	0.8924	0.6331
		4-mer	0.8146	0.8146	0.8146	0.8908	0.6292
		5-mer	0.8222	0.8222	0.8222	0.8926	0.6471
		6-mer	0.8291	0.8291	0.8291	0.8993	0.6606
		[3, 4]-mer	0.8273	0.7945	0.8600	0.8965	0.6559
		[3, 5]-mer	0.8402	0.7892	0.8912	0.9137	0.6840
		[3, 6]-mer	0.8444	0.8479	0.8410	0.9184	0.6889
		[4, 5]-mer	0.8418	0.8109	0.8727	0.9158	0.6849
		[4, 6]-mer	0.8505	0.8077	0.8933	0.9194	0.7036
		[5, 6]-mer	0.8465	0.8500	0.8431	0.9182	0.6931
		[3, 4, 5]-mer	0.8413	0.8077	0.8748	0.9149	0.6841
		[3, 4, 6]-mer	0.8428	0.8347	0.8510	0.9170	0.6858
		[3, 5, 6]-mer	0.8484	0.7987	0.8980	0.9185	0.7002
		[4, 5, 6]-mer	0.8465	0.7739	0.9192	0.9181	0.7005
		[3, 4, 5, 6]-mer	0.8497	0.8024	0.8970	0.9180	0.7026
	T.thermophila	3-mer	0.8789	0.8789	0.8789	0.9432	0.7664
		4-mer	0.8782	0.8782	0.8782	0.9436	0.7683
		5-mer	0.8801	0.8801	0.8801	0.9442	0.7677
		6-mer	0.8801	0.8801	0.8801	0.9443	0.7697
		[3, 4]-mer	0.8790	0.9530	0.8049	0.9417	0.7664
		[3, 5]-mer	0.8806	0.9349	0.8263	0.9427	0.7657
		[3, 6]-mer	0.8811	0.9547	0.8075	0.9431	0.7707
		[4, 5]-mer	0.8809	0.9352	0.8265	0.9437	0.7663
		[4, 6]-mer	0.8789	0.9405	0.8174	0.9411	0.7637
		[5, 6]-mer	0.8795	0.9542	0.8048	0.9427	0.7676
		[3, 4, 5]-mer	0.8785	0.9286	0.8285	0.9411	0.7608
		[3, 4, 6]-mer	0.8789	0.9256	0.8322	0.9426	0.7611
		[3, 5, 6]-mer	0.8800	0.9595	0.8005	0.9432	0.7698
		[4, 5, 6]-mer	0.8800	0.9588	0.8013	0.9435	0.7697
		[3, 4, 5, 6]-mer	0.8818	0.9422	0.8214	0.9442	0.7692
	Tolypocladium	3-mer	0.7638	0.7638	0.7638	0.8288	0.5307
		4-mer	0.7546	0.7546	0.7546	0.8267	0.5096
		5-mer	0.7629	0.7629	0.7629	0.8385	0.5289
		6-mer	0.7676	0.7676	0.7676	0.8452	0.5360
		[3, 4]-mer	0.7839	0.7383	0.8295	0.8599	0.5702
		[3, 5]-mer	0.7889	0.7697	0.8082	0.8668	0.5783
		[3, 6]-mer	0.6231	0.4458	0.8005	0.6979	0.2634
		[4, 5]-mer	0.7892	0.7490	0.8295	0.8703	0.5803
		[4, 6]-mer	0.7901	0.7578	0.8224	0.8693	0.5814
		[5, 6]-mer	0.7895	0.7460	0.8330	0.8676	0.5812
		[3, 4, 5]-mer	0.7854	0.7460	0.8247	0.8679	0.5725
		[3, 4, 6]-mer	0.7913	0.7353	0.8472	0.8729	0.5863
		[3, 5, 6]-mer	0.7913	0.7774	0.8052	0.8686	0.5828
		[4, 5, 6]-mer	0.7857	0.7407	0.8307	0.8675	0.5737
		[3, 4, 5, 6]-mer	0.7910	0.7413	0.8407	0.8660	0.5849
	Xoc.BL256	3-mer	0.8834	0.8834	0.8834	0.9504	0.7676
		4-mer	0.8728	0.8728	0.8728	0.9240	0.7456
		5-mer	0.8786	0.8786	0.8786	0.9496	0.7574
		6-mer	0.8823	0.8823	0.8823	0.9451	0.7647
		[3, 4]-mer	0.8736	0.8815	0.8658	0.9356	0.7474
		[3, 5]-mer	0.8817	0.8820	0.8814	0.9362	0.7633
		[3, 6]-mer	0.8850	0.8871	0.8830	0.9387	0.7701
		[4, 5]-mer	0.8813	0.8932	0.8694	0.9367	0.7629
		[4, 6]-mer	0.8852	0.8907	0.8796	0.9517	0.7704
		[5, 6]-mer	0.8820	0.8809	0.8830	0.9484	0.7639
		[3, 4, 5]-mer	0.8810	0.8965	0.8656	0.9505	0.7624
		[3, 4, 6]-mer	0.8842	0.8741	0.8944	0.9529	0.7686
		[3, 5, 6]-mer	0.8860	0.8850	0.8870	0.9528	0.7719
		[4, 5, 6]-mer	0.8836	0.8957	0.8716	0.9391	0.7675
		[3, 4, 5, 6]-mer	0.8864	0.8883	0.8844	0.9520	0.7727

Table S7. Performance of different fusion strategies on 17 benchmark datasets.

Methylation Type	Species	Strategies	ACC	SN	SP	AUC	MCC
**5hmC**	H.sapiens	Avg. Fusion	0.9480	0.9761	0.9198	0.9721	0.8973
		Fusion module	0.9501	0.9829	0.9172	0.9579	0.9021
	M.musculus	Avg. Fusion	0.9676	0.9685	0.9668	0.9856	0.9353
		Fusion module	0.9679	0.9690	0.9668	0.9827	0.9358
**4mC**	C.equisetifolia	Avg. Fusion	0.8689	0.8415	0.8962	0.9105	0.7388
		Fusion module	0.8852	0.8634	0.9071	0.9308	0.7600
	F.vesca	Avg. Fusion	0.8451	0.8665	0.8236	0.9163	0.6908
		Fusion module	0.8528	0.8847	0.8210	0.9235	0.7071
	S.cerevisiae	Avg. Fusion	0.7250	0.6421	0.8079	0.7801	0.4563
		Fusion module	0.7432	0.6997	0.7867	0.8075	0.4882
	Tolypocladium	Avg. Fusion	0.7392	0.7357	0.7427	0.8128	0.4784
		Fusion module	0.7494	0.7316	0.7672	0.8241	0.4991
**6mA**	A.thaliana	Avg. Fusion	0.8611	0.8560	0.8662	0.9356	0.7222
		Fusion module	0.8627	0.8107	0.9148	0.9379	0.7294
	C.elegans	Avg. Fusion	0.9158	0.9410	0.8907	0.9677	0.8327
		Fusion module	0.9214	0.9384	0.9043	0.9727	0.8432
	C.equisetifolia	Avg. Fusion	0.7549	0.7138	0.7959	0.8274	0.5115
		Fusion module	0.7610	0.6894	0.8325	0.8358	0.5274
	D.melanogaster	Avg. Fusion	0.9263	0.9199	0.9326	0.9740	0.8526
		Fusion module	0.9283	0.9301	0.9265	0.9740	0.8567
	F.vesca	Avg. Fusion	0.9446	0.9484	0.9407	0.9821	0.8891
		Fusion module	0.9468	0.9491	0.9446	0.9711	0.8936
	H.sapiens	Avg. Fusion	0.9126	0.9001	0.9251	0.9721	0.8254
		Fusion module	0.9126	0.9038	0.9215	0.9718	0.8254
	R.chinensis	Avg. Fusion	0.8946	0.8963	0.8930	0.9442	0.7893
		Fusion module	0.9080	0.9097	0.9064	0.9595	0.8161
	S.cerevisiae	Avg. Fusion	0.8442	0.8051	0.8833	0.9188	0.6904
		Fusion module	0.8497	0.8024	0.8970	0.9180	0.7026
	Tolypocladium	Avg. Fusion	0.7931	0.7359	0.8502	0.8713	0.5900
		Fusion module	0.7910	0.7413	0.8407	0.8660	0.5849
	T.thermophila	Avg. Fusion	0.8827	0.9471	0.8183	0.9448	0.7719
		Fusion module	0.8818	0.9422	0.8214	0.9442	0.7692
	Xoc.BLS256	Avg. Fusion	0.8839	0.8875	0.8803	0.9472	0.7679
		Fusion module	0.8864	0.8883	0.8844	0.9520	0.7727
